# Late Presentation of Persistent Left Superior Caval Vein in a Univentricular Heart with Successful Transcutaneous Occlusion Using Cera Lifetech Atrial Septal Occluder

**DOI:** 10.1155/2014/383529

**Published:** 2014-11-06

**Authors:** Iyad AL-Ammouri, Ahmad Alhourani, Ayoub Innabi

**Affiliations:** ^1^Department of Pediatrics, Faculty of Medicine, University of Jordan, Amman 11940, Jordan; ^2^School of Medicine, University of Jordan, Amman 11940, Jordan

## Abstract

We present a case of persistent left superior caval vein in a univentricular heart presenting with progressive and disabling cyanosis in a 35-year-old man eighteen years after his Kawashima operation. The vein was successfully occluded using an atrial septal occluder with significant improvement of symptoms and oxygen saturation.

## 1. Introduction

A persistent left superior caval vein with a reported incidence of 0.3% to 0.5% in the general population is considered the most common congenital venous anomaly of the thoracic venous return [1]. It has been reported in up to 12% of individuals with congenital heart abnormalities, such as septal defects, aortic coarctation, transposition of the great vessels, tetralogy of Fallot, and anomalous connections of the pulmonary veins [2–4].

The persistent left superior caval vein usually drains into the right atrium via the coronary sinus. In the rare case of coronary sinus ostial atresia, the left superior caval vein drains the coronary venous blood flow from the coronary sinus to the systemic venous circulation. These anomalies cause no shunts [1, 5]. However, in 10–20% of cases, it drains to the left atrium either via unroofed coronary sinus, or in a straight line fashion into the roof of the left atrium, or the left superior pulmonary vein [6]. It is in this kind of abnormal connection where there is shunting that it may need to be occluded or ligated. Presentation of left superior caval vein in patients following single ventricle palliation has been reported several years after surgery [7]. We report a case where this anomaly was detected 18 years after surgery and was treated successfully by cardiac catheterization.

## 2. Case Report 

A 35-year-old man with the diagnosis of mitral atresia, a double outlet right ventricle, pulmonary atresia with interrupted inferior caval vein, and azygous continuation to the right superior caval vein underwent a Blalock-Taussig shunt in early childhood and a Kawashima operation at the age of 17 at another institution. He presented to our clinic with progressive cyanosis and fatigue 18 years after his last surgical procedure. His oxygen saturation was 65%.

**Figure 1:**
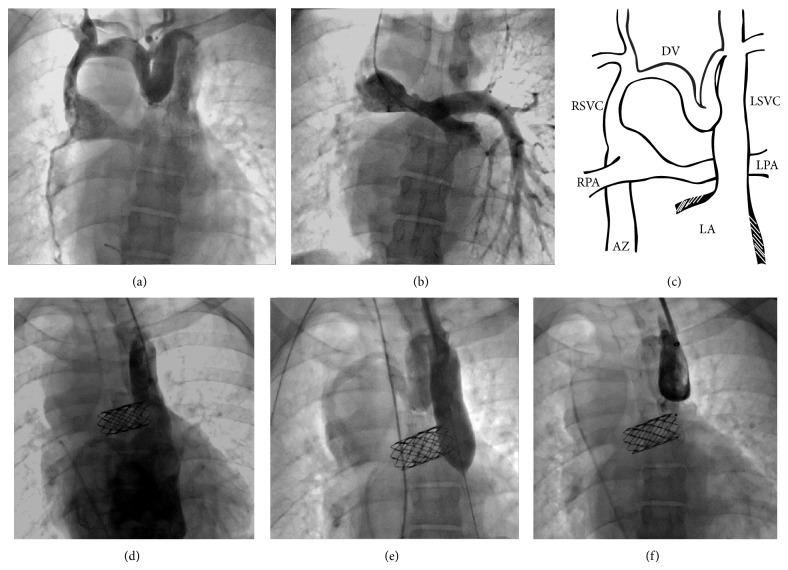
Vein angiography in a 35-year-old patient with univentricular heart 18 years following Kawashima operation. (a) Angiography through right internal jugular vein shows flow from the right superior caval vein to the left superior caval vein via a tortuous decompressing vein. (b) Angiography of the pulmonary artery shows mild narrowing of the left pulmonary artery. (c) Schematic of the venous anatomy. (d) During a separate procedure after stent placement in the left pulmonary artery, angiography through left jugular vein showing the large left superior caval draining to the left atrium. (e) Angiography during balloon test occlusion. (f) Angiography following device closure showing residual leak. (AZ: azygous vein, DV: decompressing vein, LA: left atrium, LPA: left pulmonary artery, LSVC: left superior caval vein, RPA: right pulmonary artery, and RSVC: right superior caval vein.)

Cardiac catheterization revealed left pulmonary artery stenosis and a decompressing vein from the right superior caval vein to the left superior caval vein which directly drains to the left atrium. Left pulmonary artery stenting was done via right internal jugular approach (Figures 1(a), 1(b), and 1(c)).

He underwent a second catheterization for occlusion of the left superior caval vein via left internal jugular approach. The anomalous vein measured 22 and 20 mm in AP and lateral projections, respectively. Balloon test occlusion resulted in immediate increase in oxygen saturation from 65% to 82% without drop of blood pressure. The mean pressure in the azygous vein remained at 9 mmHg; pressure in the left internal jugular vein increased to 11 mmHg and dropped within a few minutes to 10 mmHg. Angiography showed good venous collateral drainage.

The largest available vascular plug, 24 mm in diameter, failed to completely occlude the vessel, which was successfully occluded using a 24 mm ASD occluder (Cera Lifetech, China) via a 12 Fr sheath. Upon completion of the procedure the patient had oxygen saturation of 80%, left internal jugular pressure of 10 mmHg, and right superior caval vein and pulmonary artery pressure of 9 mmHg.

After occlusion, the patient had significant improvement of his exercise intolerance and fatigue. Follow-up hemodynamic study 12 months later showed no increase in venous pressure, no development of venous collaterals, and no decrease in his oxygen saturation.

## 3. Discussion

Generally, a persistent left superior caval vein draining into the right atrium through a coronary sinus has no hemodynamic consequence but draining into the left atrium creates a small obligatory right-to-left shunt [4]. Although this drainage to the left atrium is commonly asymptomatic, it may cause in some cases hypoxemia, cyanosis, clubbing of the nail bed, and some major complications like paradoxical embolization [4, 6, 8]. This may be more exaggerated in patients with univentricular hearts where the systemic venous pressure is always higher than left atrial pressure.

In our patient the diagnosis of a persistent left superior caval vein was made after presentation of progressive and symptomatic cyanosis 18 years after his cavopulmonary anastomosis. From the previous records of his operation at another institution it was unclear if the anomalous vein was identified. Since it is draining directly into the roof of the left atrium and the communicating vein to the right superior caval vein was long and tortuous, we tested by balloon occlusion to see if there is any effect on the cardiac output or the venous pressure before permanently occlude the vein. Balloon test occlusion has been well described before transcatheter occlusion of systemic venous anomalies [9].

In our patient there was no significant change in the venous or arterial pressure with significant improvement of oxygen saturation indicating a significant improvement of pulmonary blood flow. Because of the geometry of the vascular plug and the absence of retention discs in its design as well as the relatively large size of the vein, closure was not successful using vascular plug. Use of atrial septal occluder enabled secure placement due to the presence of retention discs. This was reported in previous reports as an off-label use of the atrial septal occlude [10–12].

## 4. Conclusion

Patients with single ventricle physiology should have regular follow-up and surveillance for right to left shunt, which can result in significant and progressive desaturation and deterioration of exercise tolerance. With the new and multiple selection of devices available in the recent years it has been possible to occlude many of the right to left shunts percutaneously.
